# Generic cardiology drug prices: the potential benefits of the Marc Cuban cost plus drug company model

**DOI:** 10.3389/fphar.2023.1179253

**Published:** 2023-08-22

**Authors:** Aparna Narendrula, Jacob Lang, Elias Mossialos

**Affiliations:** ^1^ Department of Medicine, NYU Grossman School of Medicine, New York City, NY, United States; ^2^ NewYork-Presbyterian/Weill Cornell Medicine, New York, NY, United States; ^3^ Department of Health Policy, London School of Economics and Political Science, London, United Kingdom

**Keywords:** cardiology, generic pharmaceuticals, drug prices, health policy, United States

## Abstract

**Introduction:** Generic pharmaceuticals account for the majority of the $359 billion US pharmaceutical market, including for cardiology drugs. Amidst a lack of price transparency and administrative inefficiencies, generic drug prices are high, causing an undue burden on patients.

**Methods:** We identified the 50 most used generic cardiology drugs by volume per the 2020 Medicare Part D spending data. We extracted cost per dose of each drug from the Marc Cuban Cost Plus Drug Company (MCCPDC) website and estimated the aggregate cost savings if MCCPDC were employed on a national scale by calculating the difference between this cost and Medicare spending.

**Results:** Medicare spent $7.7 billion on the 50 most used generic cardiology drugs by volume in 2020 according to Medicare Part D data. Pharmacy and shipping costs accounted for a substantial portion of expenditures. Per our most conservative estimate, $1.3 billion (17% of total) savings were available on 16 of 50 drugs. A slightly less conservative estimate suggested $2.9 billion (38%) savings for 35 of 50 drugs.

**Discussion:** There is enormous potential for cost savings in the US market for generic cardiology drugs. By encouraging increased competition, decreasing administrative costs, and advocating for our patients to compare prices between the MCCPDC and other generic pharmaceutical dispensers, we have the potential to improve access to care and corresponding outcomes for cardiology patients.

## 1 Introduction

Pharmaceutical drug costs account for a substantial proportion of US health expenditures, with $359 billion spent on pharmaceuticals in 2020. (iqvia, 2021). High administrative costs, a lack of bargaining power by multiple insurance agents, the presence of pharmaceutical intermediaries, and limited price transparency contribute to high generic drug prices. (brookings, 2020). Such high prices impair patient access to medications and provide a substantial challenge to cardiology patients and providers.

The generic pharmaceutical market is convoluted by complex business practices and a lack of price transparency, as well negotiations between payers/insurers, drug manufacturers, providers, and patients. The US pharmaceutical market is so complex that intermediaries, including pharmaceutical benefit managers (PBMs) were introduced to help the flow of finances. However, incomplete evidence surrounds our understanding of the impact of such intermediaries. Some scholars believe that they may play a role in increased drug prices that impede patient access to medications. (Dabora et al., 2017; Schulman and Dabora, 2018; Seeley and Kesselheim, 2019).

PBMs and intermediaries have substantial power to set the cost of pharmaceutical drugs, however these actors are often considered to be profit-driven, rather than driven by patient interest. This contrasts with countries with government-controlled insurance systems, where PBMs have little to no presence in influencing the prices of drugs. In the United States, 54.3% of patients had employer based coverage, 18.4% had Medicare, and 8.3% had no insurance in 2021, with rising prevalence of high-deductible health plans. (Keisler-Starkey, 2022). Many of these plans include large amounts of cost-sharing, where patients would be required to pay out of pocket for their cardiology medications before their deductible is met.

One in eight patients with atherosclerotic cardiovascular disease report nonadherence to medications because of cost. (Khera et al., 2019).The average patient with atherosclerotic disease spends $2000, while a heart failure patient spends $5,000 out of pocket (OOP) per year in addition to insurance premiums, with pharmaceutical costs being the main driver of costs to patients. (Slavin et al., 2021). This kind of financial burden on patients has been shown to cause financial distress and is negatively associated with patient access to life-saving cardiology drugs. It has also been linked to worse outcomes including cardiovascular hospitalizations, mortality, and acute myocardial infarction. (Slavin et al., 2021).

Alternative solutions to address rising generic drug costs have been at the forefront of drug pricing discussions in recent year. One such approach is the Mark Cuban Cost Plus Drug Company (MCCPDC), which aims to improve price transparency and decrease administrative inefficiencies in the generic drug market by bypassing pharmaceutical intermediaries, who account for a large proportion of the exorbitant drug costs seen by patients. (costplusdrugs, 2020).

MCCPDC was founded in 2022. They advertise a 15% markup, $3 pharmacy fee, and $5 shipping cost for a growing list of off-patent medications, many of which are employed by cardiac providers. (costplusdrugs, 2020). The aim of this is study is to assess whether assess whether Medicare-scale purchasing of cardiology medications through the MCCPDC model would lead to cost savings.

## 2 Methods

We identified the 50 most used generic cardiology drugs by volume per the 2020 Medicare Part D spending data, which was the most recently available data at the time of the analysis (December 2022). Medicare Part D provides its voluntary beneficiaries with financial aid through outpatient prescription drug coverage. The publicly available Medicare dataset employed in this study shares drug spending metrics related to prescription claims for these beneficiaries. We first identified the total spending in 2020 per drug for each of the 50 included drug from the Medicare Part D dataset.

As we aimed to estimate the total expenditure of an equivalent pharmaceutical volume with MCCPDC prices as opposed to Medicare Part D spending per drug, which included both generic and brand-name dispensing of each off-patent drug, we identified the total dosage units dispensed in 2020 for each drug.

To provide a conservative estimate of cost savings, we selected MCCPDC pricing for the highest dosage available for each drug. (Cortese et al., 2022). Unit prices were higher in the 30- than the 90-day supply for all drugs and included the 15% markup, $3 pharmacy fee, and $5 shipping cost advertised by the MCCPDC.

We determined the total market cost per drug by multiplying 2020 Medicare Part D total dosage units purchased by MCCPDC price, then subtracted this from total 2020 Medicare Part D spending per drug to estimate potential cost savings. (Lalani et al., 1073).

## 3 Results

**FIGURE 1:**
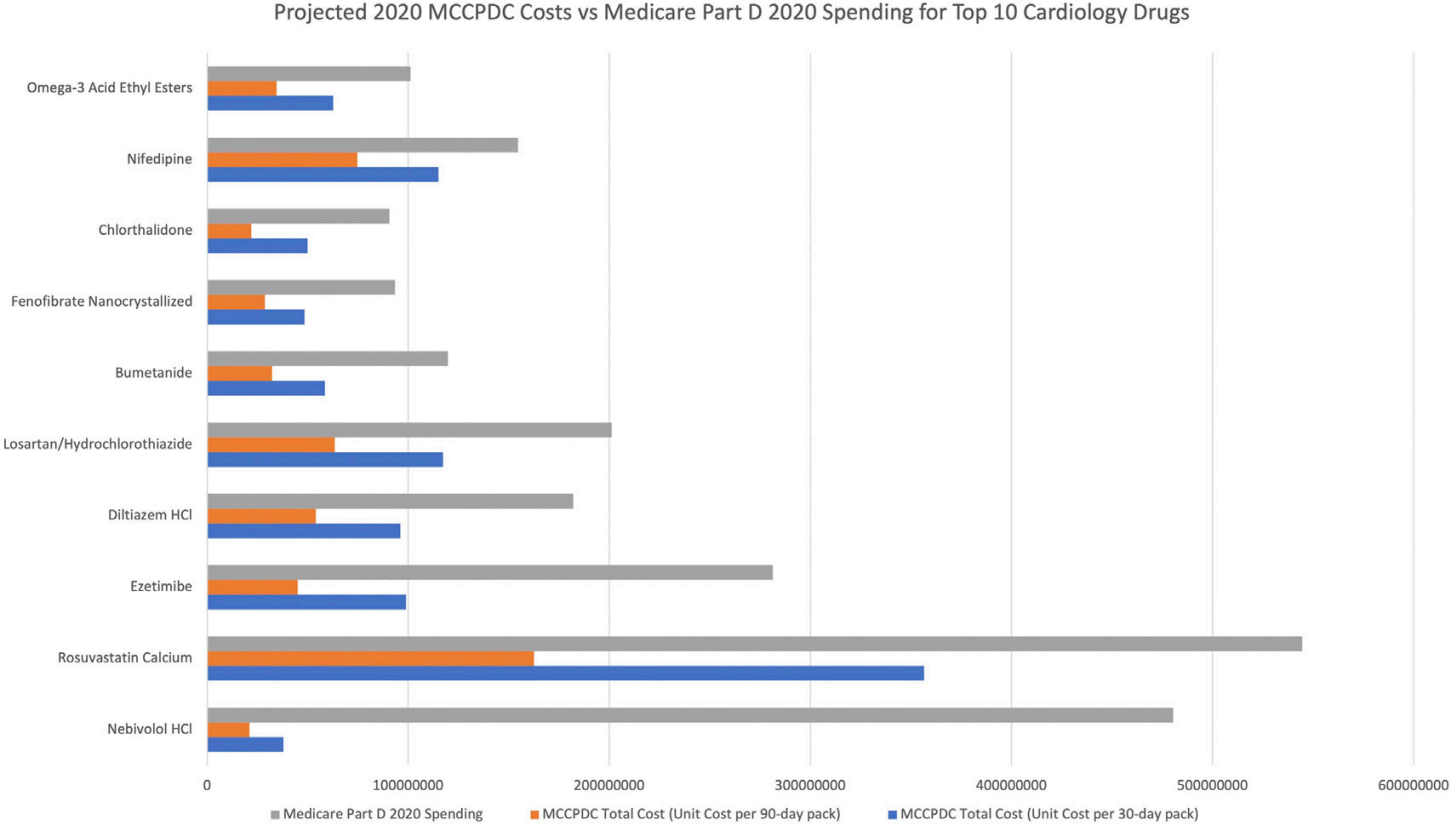
Projected spending per drug for Medicare Part D 2020 volume based on Medicare Part D 2020 pricing compared to MCCPDC pricing (both 90 and 30 day pricing).

Medicare spent $7,714,270,960 on the 50 most used generic cardiology drugs by volume according to 2020 Medicare Part D. If unit price was based on the MCCPDC 30-day price including shipping for the highest dosage, $1,338,063,146 (17% of total) savings were available on 16 of 50 drugs. Pharmacy and shipping costs accounted for 44%–93% of drug costs, except for amiodarone (21%) (Table 1). If unit price were based on 90-day supply, there would be $2,926,054,705 (38%) savings for 35 of 50 drugs. Based on 30-day prices, nebivolol ($442,412,458), rosuvastatin ($188,088,703), and ezetimibe ($182,413,233) demonstrated the highest potential cost savings (Figure 1).

**TABLE 1:** Estimated Savings with MCCPDC Prices for the 50 Most Dispensed Generic Cardiology Drugs by Medicare Part D 2020 (data accessed 12/22/2022).

Generic name	Total dosage units, in millions	Percentage of 30-day based price attributable to pharmacy and shipping fees (%)	Estimated savings per MCCPDC 30 Day prices in millions	Percentage of 90-day based price attributable to pharmacy and shipping fees (%)	Estimated savings per MCCPDC 90 Day prices in millions	Total medicare part D 2020 spending in millions
Nebivolol HCl	96	67	442	41	459	480
Rosuvastatin Calcium	1091	82	188	60	382	545
Ezetimibe	303	82	182	60	236	281
Diltiazem HCl	236	66	86	39	128	182
Losartan/Hydrochlorothiazide	304	69	84	43	138	201
Bumetanide	148	67	61	41	87	120
Fenofibrate Nanocrystallized	111	61	45	34	65	93
Chlorthalidone	158	84	41	64	69	91
Nifedipine	227	53	40	27	80	155
Omega-3 Acid Ethyl Esters	158	67	39	41	67	101
Valsartan	176	56	34	30	65	118
Digoxin	118	61	24	34	45	76
Fenofibrate	143	44	23	21	48	109
Olmesartan Medoxomil	159	60	21	33	50	92
Valsartan/Hydrochlorothiazide	111	47	18	23	37	80
Verapamil HCl	111	79	11	56	30	48
Doxazosin Mesylate	180	67	0	41	32	71
Irbesartan	172	51	−6	25	25	85
Enalapril Maleate	236	79	−8	56	34	71
Labetalol HCl	154	58	−10	32	17	60
Terazosin HCl	148	67	−18	41	8	40
Sotalol HCl	137	67	−19	41	6	36
Gemfibrozil	123	73	−19	47	3	26
Amlodipine Besylate/Benazepril	129	55	−20	29	3	43
Metoprolol Succinate	1883	73	−23	47	312	668
Ramipril	167	84	−25	64	4	28
Isosorbide Mononitrate	336	73	−28	47	32	95
Triamterene/Hydrochlorothiazid	248	79	−36	56	8	47
Lovastatin	286	90	−40	75	11	45
Propranolol HCl	275	61	−45	34	4	75
Benazepril HCl	211	73	−49	47	−12	28
Torsemide	188	52	−52	26	−18	46
Warfarin Sodium	513	84	−53	64	38	109
Spironolactone	425	69	−80	43	−4	84
Clonidine HCl	410	84	−91	64	−18	39
Pravastatin Sodium	809	69	−94	43	50	218
Clopidogrel Bisulfate	840	79	−98	56	51	185
Amiodarone HCl	151	21	−115	8	−89	75
Lisinopril/Hydrochlorothiazide	544	82	−118	60	−21	59
Atenolol	665	87	−127	69	−9	77
Hydralazine HCl	784	75	−176	50	−36	104
Hydrochlorothiazide	1404	90	−304	75	−55	112
Simvastatin	1484	79	−313	56	−49	186
Losartan Potassium	2172	79	−368	56	18	364
Furosemide	1588	79	−387	56	−105	147
Carvedilol	1874	87	−423	69	−90	151
Atorvastatin Calcium	4048	84	−435	64	284	847
Metoprolol Tartrate	2074	90	−475	75	−106	141
Amlodipine Besylate	3032	93	−562	82	−23	307
Lisinopril	2771	84	−606	64	−114	271

## 4 Discussion

Using the MCCPDC model for drug pricing, we estimate at least $1.3 billion (17%) of cost savings for the 16 of the top 50 cardiology drugs per Medicare Part D 2020 volume. By demonstrating the potential benefit of market entry by MCCPDC, we show that there is significant potential for cost-savings in the generic cardiology medication market. Our findings supplement previously published articles showing similar potential for cost savings with the MCCPDC model in the overall, urologic, and oncology generic drug markets. (Cortese et al., 2023; Cubanksi et al., 2023).

Generics have long been promoted for their ability to contain costs, as evidenced by 90% of dispensed US prescriptions being generics in 2020, up from 78% in 2010. (iqvia, 2021). Our findings are consistent with evidence that generic market entry does not necessarily lead to the lowest price possible, because a substantial portion of generic drug costs are attributable to pharmacy dispensing costs, payments to intermediaries, and profit, rather than the cost of production. (brookings, 2020). The MCCPDC model aims to reduce intermediary-related costs and directly negotiate lower prices since it employs its own PBMs. They partner with the TruePill mail-order pharmacy to supply medications, as they are currently building the infrastructure to manufacture generic drugs in Dallas, Texas. (costplusdrugs, 2020).

Pharmaceutical intermediaries include PBMs and distributors, with $115 billion paid by manufactures to third parties in 2015. (Dabora et al., 2017). PBMs are intermediaries who negotiate payments between manufacturers aiming to obtain a large market share to maximize profits, and insurers aiming to minimize spending to maximize profits. (Schulman and Dabora, 2018; Seeley and Kesselheim, 2019). PBMs use mechanisms such as payment negotiations and manufacture rebates to influence formulary placement and pharmaceutical spending. (Schulman and Dabora, 2018; Seeley and Kesselheim, 2019). Recently, there have been large scale increases in payments from manufactures to intermediaries. (Schulman and Dabora, 2018).

PBMs hold a pivotal position within the pharmaceutical market; however, their ability to create profit revolves around the maximization of rebates, since their profits are linked to a portion of these rebates. Consequently, their expertise in navigating the intricate financial dynamics of the generic drug market in the United States, coupled with their profit-driven motives, might contribute to the escalation of drug prices (brookings, 2020). Some believe that this misalignment of interests with patient wellbeing results in a lack of transparency and exorbitant medication costs within the United States (Schulman and Dabora, 2018; Seeley and Kesselheim, 2019). However, there is a gap in evidence-based knowledge pertaining to the financial effects of PBMs on patients (Rosenthal et al., 2023).

Our findings have implications within the framework of the Inflation Reduction Act of 2022, specifically regarding the provisions related to prescription drugs (Congress, 2022). This legislation is designed to address the issue of escalating drug prices through various measures, such as facilitating federal government negotiations to lower Medicare Part D drug prices, implementing mandatory rebates for manufacturers if prices exceed the inflation rate, reducing cost-sharing obligations for patients, and introducing a $2000 out-of-pocket spending limit for Part D by 2025. Notably, these approaches align with the strategies employed by MCCPDC and Amazon in the private sector, all aimed at the common objective of reducing prices for generic drugs (Cortese et al., 2023; Cubanksi et al., 2023). Other limitations include that we assume purchasing of the highest dosage of each medication, therefore potentially underestimating cost savings. For example, our estimate of the market cost of metoprolol succinate assumes all units purchased are 200mg, despite likely utilization of lower and less expensive dosages in a significant proportion of the population. Also, the Medicare Part D spending data may overestimate United States spending on prescription drugs since it does not account for price concessions or manufacturers’ rebates.

In conclusion, we demonstrate that Medicare is overpaying for off-patent cardiology drugs, and that there is potential for substantial market savings for certain cardiology drugs using the private-sector MCCPDC model, in large part by bypassing intermediaries. Payers and clinicians should encourage patients to choose generic medications, including through the MCCPDC, where cost savings are present.

The potential for cost savings through innovative private or public sector approaches is important to encourage in the cardiology field. It is crucial that cardiology providers and policymakers continue to use economic creativity and create solutions to address inefficiencies that impede patient access to cardiology care. (Ali et al., 2022).

## Data Availability

Publicly available datasets were analyzed in this study. This data can be found here: https://costplusdrugs.com/ and https://data.cms.gov/summary-statistics-on-use-and-payments/medicare-medicaid-spending-by-drug/medicare-part-d-spending-by-drug.
